# Supplementary material for: Pharmacokinetics of Novel Crystalline Buntanetap in Mice, Dogs, and Humans
Source: Biomolecules. 2025 Sep 10;15(9):1299. doi: 10.3390/biom15091299 (PMC12467374; doi:10.3390/biom15091299)

Figure S1: XRPD data collection parameters.

**Data Collection Parameter**	Value
**Geometry**	Bragg–Brentano
**Tube Anode**	Cu
**Tube Type**	Long Fine Focus
**Tube Voltage (kV)**	40
**Tube Current (mA)**	44/45/50
**Detector**	D/teX Ultra 250 (XR1/XR3/XR5) HyPix-3000 (XR4)
**Monochromator**	Ni foil Cu Kβ Filter
**Incident Slit (°)**	1/3
**Receiving Slit 1 (mm)**	18
**Receiving Slit 2 (mm)**	Open/20
**Start Angle 2θ (°)**	2
**End Angle 2*θ* (°)**	40
**Step Size (°)**	0.02
**Scan Speed (°/min)**	6
**Spinning (rpm)**	11
**Sample Holder**	Low-background Si

Figure S2: MicroED analysis instruments parameters.

**Powder X-ray diffractometer**	D8 Advance (Bruker)
**Polarized-light microscope**	Axio Scope 5 POL (Ziess)
**CryoEM**	Talos F200C (200 kV, Thermo Fisher Scientific)
**Detector**	Ceta-D (CMOS, Thermo Fisher Scientific)
**Data collection software**	EPU-D (Version 1.18.0.6930, Thermo Fisher Scientific)
**CryoEM grid**	Holey Carbon EM grid (Cu300 mesh *R*1.2/1.3, QUANTIFOIL)
**Cryogenic sample holder**	Model 626 (Gatan)
**Consumables**	Glass slide, ethanol and kimwipes

Figure S3. Sample collections schedule in mice. A total of 15 mice for each form of buntanetap (Form A and Form B) was used in triplet groups to reach the n=3 of sample replicates per time point for brain, CSF, and plasma.

Triplet mouse groups			Day	Time point (h)	Brain	CSF	Plasma
#1	#2	#3	1	0.25			X
				0.5	x	X	X
#4	#5	#6	1	0.75			X
				1	X	X	X
#7	#8	#9	1	1.25			X
				1.5			X
				1.75			X
				2	X	X	X
#10	#11	#12	1	3			X
				4			X
				5			X
				6	X	X	X
#13	#14	#15	1	8			X
				10			X
				12	X	X	X

Figure S4. Dog diet. Main ingredients, testing method, and compliance statement. The diet was received by the Cooperative Medical Biological Engineering Co., LTD in Jiangsu Province.

**Test Item**	**Unit**	**Testing Method**	**Test Result**	**Quantitative Limit**	**Limit Value**	**Compliance Statement**
Crude Protein	g/kg	GB/T 6432-2018	252.1	-	≥200	Compliant
Moisture	g/kg	GB/T 6435-2014	80.0	-	≤100	Compliant
Crude Ash	g/kg	GB/T 6438-2007	69.0	-	≤90	Compliant
Crude Fiber	g/kg	GB/T 6434-2022	32.0	-	≤40	Compliant
Total Phosphorus	g/kg	GB/T 6437-2018	7.80	0.06	5-8	Compliant
Crude Fat	g/kg	GB/T 6433-2006 B	86.0	-	≥45	Compliant
Calcium	g/kg	GB/T 6436-2018 3	9.49	-	7-10	Compliant
Lysine	g/kg	GB/T 18246-2019	13.65	0.4	≥7.1	Compliant
Cystine	%	GB/T 18246-2019	0.41	0.01	-	-
Methionine	%	GB/T 18246-2019	0.62	0.01	-	-
Aflatoxin B1	μg/kg	GB 5009.22-2016	ND	0.30	≤20.0	Compliant
Arsenic (As)	mg/kg	GB 5009.11-2014	0.17	0.010	≤0.7	Compliant

Figure S5: Capsule composition used in dog diet. The capsules were manufactured under controlled conditions in accordance with cGMP to guarantee that capsules conform to the highest quality standard.

**Characteristics (Test)**	**Specification**	**Result**	**Compliance Statement**
Moisture (loss on drying)	13-15% w/w	13-14%	Compliant
Disintegration time	≤15min	5min	Compliant
Average weight	0.69 g +/-10%	0.72g	Compliant
Total Aerobic microbial count	≤1000 cfu/g	160 cfu/g	Compliant
Total combines Yest and Mold count	≤100 cfu/g	<10 cfu/g	Compliant

Figure S6: Comparison of XRPD patterns of Form A and Form B. Dissolving a high amount of Form A (~200 mg/ml) in water for an attempted slow evaporation resulted in spontaneous recrystallization of a new form designated Form B.

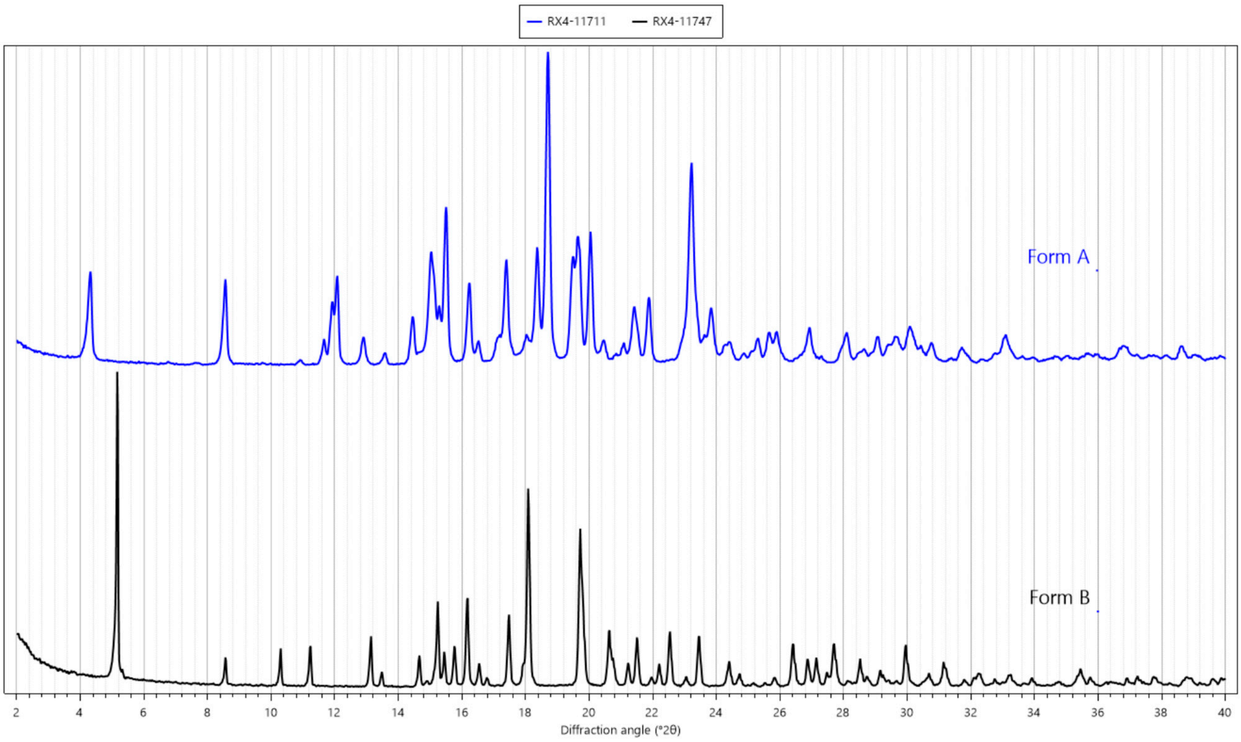

Figure S7: DSC (green) and TGA (blue) traces of buntanetap Form A and Form B. For Form A, DSC analysis shows a broad minor endotherm at 81°C, followed by a major endotherm at 146°C, likely attributable to the melting of the material. TGA analysis reveals a weight loss of 0.84% by 125°C, followed by onset of decomposition at 153°C. For Form B, TGA trace shows a 7.1% weight loss starting at 76°C coupled with a major endotherm at 93°C in its DSC trace, suggesting that Form B is a dihydrate possessing 2 moles of water per molecule of salt.

Form A

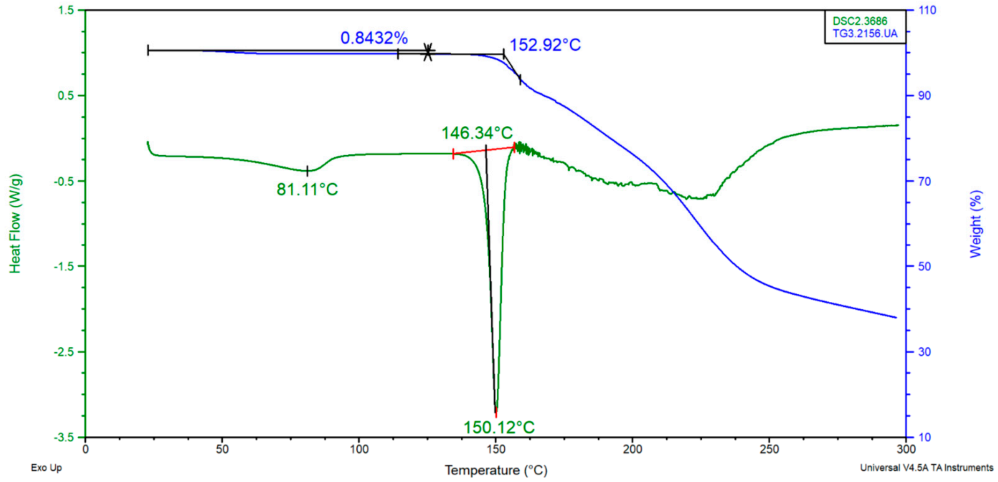

Form B

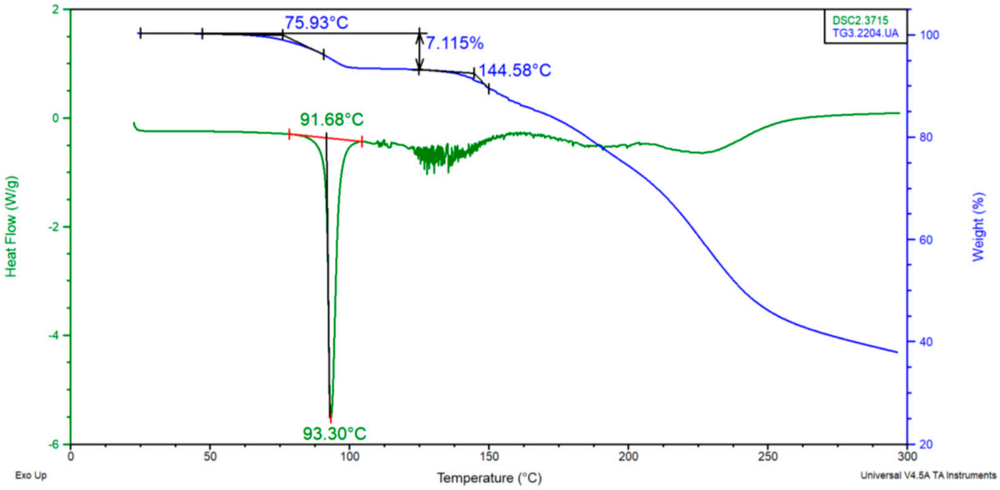

Figure S8: Plasma concentration of Forms A and B and its metabolites (N1- and N8-norbuntanetap) in male and female dogs. No sex-related significant differences were observed for both forms for main parameters (t-test): Buntanetap - Cmax (p=0.695), T1/2 (p=0.703), Tmax (p=0.526), AUC0-last (p=0.545); N1-norbuntanetap - Cmax (p=0.778), T1/2 (p=0.680), Tmax (p=0.603), AUC0-last (p=0.430); N8-norbuntanetap – Cmax (p=0.686), T1/2 (p=0.959), Tmax (p=0.567), AUC0-last (p=0.552).

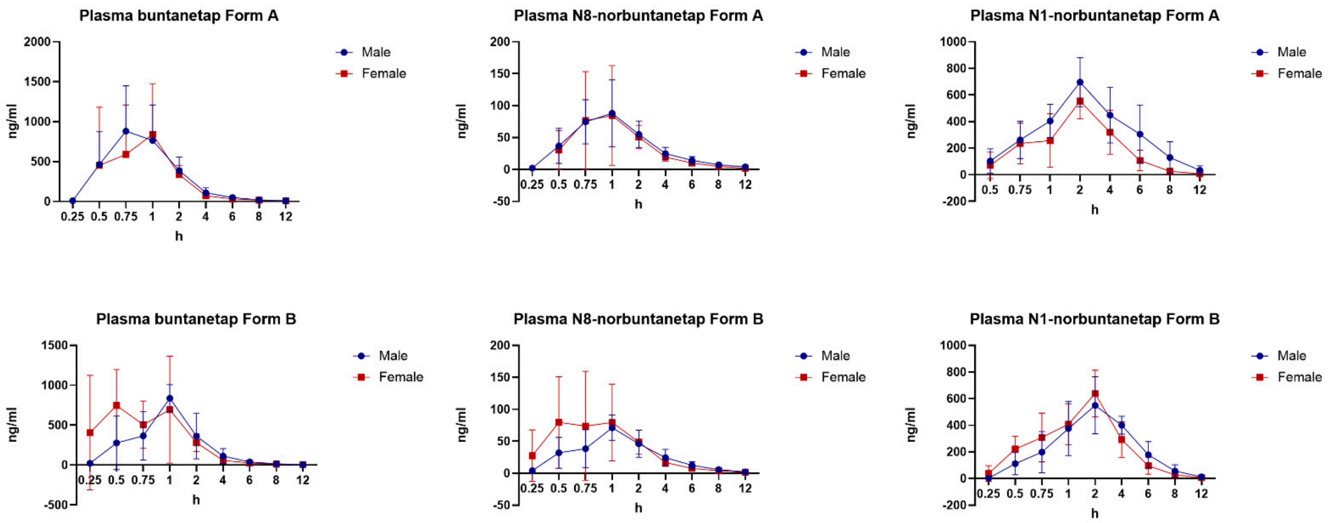

Supplement: Supplementary file 1 [file biomolecules-15-01299-s001.zip › biomolecules-3784458-supplementary.pdf]
